# Certainty and systematicity of practice-derived evidence matter for its relative importance in professional decision-making: Survey results on the role of proven experience in Swedish medicine, nursing, OT, dentistry, and dental hygiene

**DOI:** 10.1016/j.ijnsa.2022.100074

**Published:** 2022-03-17

**Authors:** Johannes Persson, Annika Wallin, Barry Dewitt, Lena Wahlberg

**Affiliations:** a Department of Philosophy. Lund University, Lund, Sweden; b Lund University Cognitive Science, Department of Philosophy, Lund University, Lund, Sweden; c Department of Engineering & Public Policy, Carnegie Mellon University, Pittsburgh, US, and Division of Medical Ethics, Lund University, Lund, Sweden; d Department of Law, Faculty of Law, Lund University, Lund, Sweden

**Keywords:** Clinical expertise, Clinical judgment, Evidence-based medicine, Proven experience, Medical decision-making, Epistemology, Sweden

## Abstract

**Background:**

High-quality healthcare decisions need to balance input from science and clinical practice. When two sources of evidence — such as scientific and practice-derived evidence — are compared, integrated, or need to stand-in for one another, they need to be comparable on similar dimensions. Since 1891, Swedish physicians have been operating under a legal requirement to base their healthcare decisions on science and “proven experience” (approximately clinical expertise), and today all healthcare personnel in Sweden fall under this legal requirement.

**Objectives:**

We investigated the dynamics between these two kinds of evidence with respect to importance, systematicity, and certainty by studying Swedish healthcare professionals.

**Design:**

Survey to professionals; document studies of political discourse.

**Method:**

In this study, a survey was sent to simple random samples of Swedish professionals in medicine, nursing, occupational therapy, dentistry, and dental hygiene, asking about the roles of science and proven experience in medical decision making. Outcome measures were how important, certain, and systematic science and proven experience are for successful medical decision making.

**Participants:**

The sampling frame was each profession's most recent occupational registry accessed by the Swedish federal statistical agency. 3500 surveys were distributed. 1626 surveys were returned. 26 participants were removed prior to analysis (exclusion criteria: more than one profession indicated, missing certificate, and mistake in stratum). The final sample consisted of 295 physicians, 300 nurses, 365 occupational therapists, 339 dentists, and 301 hygienists. 162 responses in questions used as variables in the analyses were either uninterpretable or empty; those were replaced with the modal response for a given participant's profession on a given question.

**Results:**

In the study, proven experience's perceived importance for clinical decision making is positively correlated with its certainty and systematicity, and an increased certainty and systematicity is positively correlated with a diminished difference in importance between science and proven experience for almost all professions surveyed in this study.

**Conclusions:**

Proven experience has an evidentiary role in clinical decision making, and this role depends in part on its certainty and systematicity. Notably, this makes the EBM-based perspective that practice-derived knowledge is primarily of implementation value less plausible.

What is already known

• The evidentiary value of different kinds of evidence has been formulated in guidelines such as, for instance, GRADE.
• The role of clinical experience and judgment in evidence based healthcare has been discussed.

What this paper adds

• An empirical analysis of the perceived importance, certainty, and systematicity of practice-derived evidence in five healthcare professions.
• An analysis of the dynamic between scientific and practice-derived evidence, as manifested in the Swedish requirement to take science and qualified – beprövad, proven – practice-derived knowledge into account in professional and curricular development.

Evidence-based medicine (EBM) has received a lot of attention in the last decades and has been the subject of widespread curricular development and continuing medical education. EBM is to a degree a normative notion. It has promoted the use of scientific evidence but struggled with the role and importance of practice-derived evidence – perhaps even downplayed it in its early formulations (Haynes et al. 2002). Practice-derived evidence was considered important from the start but primarily when systematic, research-based evidence is lacking (EBMWG 1992). In contrast, Sweden has since long one of the most explicit formulations of the requirement to take both scientific evidence and practice-derived knowledge into account in professional decision making and education. A specific concept, *Vetenskap och Beprövad Erfarenhet (VBE) – which translates to science and proven experience* – captures the requirement. VBE is clearly a normative notion, and since 1891 also a legal notion.

It often remains an empirical question if and if so to what extent such normative notions affect practice. For instance, when both scientific evidence and practice-derived knowledge are required, as in VBE, does this make the two kinds of evidence more commensurable? Does it affect the content of the two in such a way that they become more and more overlapping (as a convergence hypothesis would have it, see e.g Persson 2021), or do they rather become complementary (i.e. divergent)? And does the perceived importance of practice-derived knowledge come to depend on its certainty and systematicity – characteristics we typically attribute to scientific evidence, and which the EBM movement (see e.g EBMWG 1992) has often thought distinguishes scientific evidence from clinical experience?

After a brief introduction to the balancing problem of scientific evidence and practice-derived evidence in EBM and VBE, this paper attempts to highlight some of the dynamics between the two kinds of evidence in five healthcare professions: medicine, nursing, OT, dentistry, and dental hygiene.

## 1 EBM's balancing problem

To achieve the right balance between input from science and practice to decision-making in medical and health care is an intriguing problem, especially salient if one accepts the evidence-based medicine framework (EBM), the currently dominant view of both practicing and teaching medicine and healthcare. Ever since its conception the *balancing problem* has haunted EBM and led to a makeover of its original ambitions. The expression “evidence-based medicine” was coined first by Gordon Guyatt, in 1990, when arguing for an alternative, supposedly better, way of making clinical decisions in healthcare (Daly 2005). The group of clinical epidemiologists he belonged to wanted to position themselves against the received practice of medical education, which they thought bred authority-dependent, uncritical physicians who trusted clinical experience too much. There was thus from the beginning of EBM an inbuilt tension between scientific evidence and practice-derived knowledge. The term “evidence-based medicine” itself was stipulatively defined in 1991 as “the application of scientific method in determining the optimal management of the individual patient” (Guyatt 1991). As a *source* of evidence, clinical expertise was downplayed in EBM (see e.g EBMWG 1992). However, the need for healthcare personnel to *appraise* evidence was highlighted:

“A new paradigm for medical practice is emerging. Evidence-based medicine de-emphasizes intuition, unsystematic clinical experience and pathophysiologic rationale as sufficient grounds for clinical decision making and stresses the examination of evidence from clinical research. Evidence based medicine requires new skills of the physician, including efficient literature searching and the application of formal rules of evidence evaluating the clinical literature.” (EBMWG 1992, 2420)

Later, Sackett et al. (1996, 71) put their thoughts in a more cautious way:

“The practice of evidence based medicine means integrating individual clinical expertise with the best available external clinical evidence from systematic research. By individual clinical expertise we mean the proficiency and judgment that individual clinicians acquire through clinical experience and clinical practice.”

The de-emphasizing parts are more concealed in contemporary EBM (see e.g Heneghan 2015); and “best evidence” might in special cases comprise more than evidence from clinical research (see also e.g Haynes et al. 2002.). “Real” EBM, Greenhalg et al. (2014) argues, is characterized by expert judgment rather than mechanical rule-following.

At the same time, instruments such as GRADE (The Grading of Recommendations Assessment, Development and Evaluation) were developed with the ambition to provide “a common, sensible and transparent approach to grading quality (or certainty) of evidence and strength of recommendations” (https://www.gradeworkinggroup.org). That different organizations now use the GRADE approach makes it less obvious that it is valuable that healthcare personnel appraise evidence on an individual basis. There is thus a slight move away from EBM's original idea that each physician should critically and systematically evaluate the evidence in a scientific way and integrate it with personal experiences and patients’ preferences, and towards a position where each doctor should be an “evidence user” with “a readiness to identify evidence-based sources which summarize the evidence for them” (Gyatt, as interviewed in Daly (2005), 91).^1^ At the same time, instruments such as GRADE, point to the possibility that different kinds of evidence can be compared on several dimensions – and thus that they share important features and are commensurable. The evidential hierarchies built into such instruments tend to emphasize scientific evidence, when such evidence is of high quality.

## 2 Science and proven experience

The slightly problematic journey of EBM, as far as the weighing of evidence from professional practice and science is concerned, is one reason why the Swedish requirement of science and proven experience is of general interest.

The demand for science and proven experience has been put to use in Swedish medicine for a very long time (see e.g Eklöf 2000.), but the first time it is used in a legal context – a Royal Decree governing the work of medical doctors – is in the 1890s:

“Each physician, whether he be an employee at an institution or an independent practitioner of the medical profession, is obliged, as his chief duty, to deliver such counsel, and, as far as circumstances permit, to extend such therapeutic endeavors, to every patient under his care as are necessitated by the patient's condition and as are consonant with science and proven experience.” (Pontin 1891, § 59, our translation)

Since 1994, not only doctors but all health and medical care personnel in Sweden have been under an obligation to conduct their work in accordance with this standard. The conception is explicit since it is demanded that one always takes science and qualified – *beprövad, proven*^2^ *–* practice-derived knowledge into account in professional and curricular development. It is precise since it is not practice-derived beliefs in general (such as all forms of personal experience) but proven experience in particular one is required to take into account. Today, the Swedish Patient Act (2014:821) states that “The patient shall be delivered competent and careful health and medical care, which is of good quality and consonant with science and proven experience”, and the Patient Safety Act (2010:659) prescribes that “healthcare and medical personnel shall conduct their work in a way that is consonant with science and proven experience”. The Swedish Higher Education Act (1992:1434) states that “the Government shall establish higher education institutions for the provision of courses and study programmes based on scholarship or artistic practice and on proven experience.”

### 2.1 How professionals understand proven experience

We know very little about the actual balancing of scientific evidence and practice-derived evidence that takes place within the professions governed by the legal notion of VBE – or in higher vocational training (even if the Swedish National Agency for Education has ambitiously tried to make the notion more precise in a series of communications, such as Skolverket 2014). Indeed, until recently we have known very little about what more exactly proven experience is taken to be by the professionals, and we still do not know how it is supposed to relate to science and scientific evidence. The legal acts that use the notion are identical in respect to how they define ‘science and proven experience’. That is: Not at all (see e.g Wahlberg and Persson 2017.). The role of proven experience has not been spelled out.

**Fig. 1:**
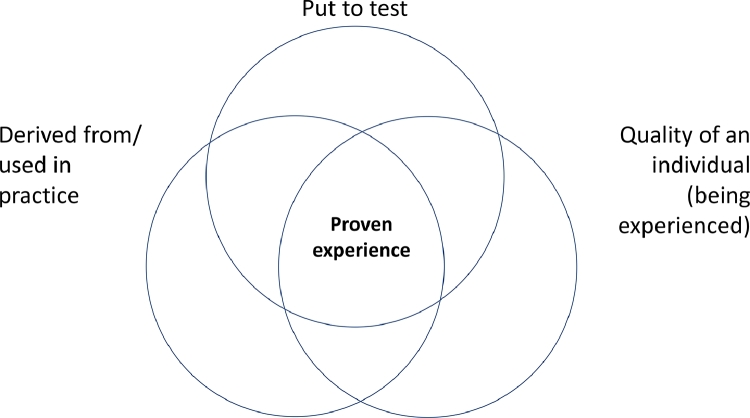
Three dimensions of proven experience. Note that uses of proven experience outside the intersection of the three dimensions occur; none of the dimensions is a necessary component of the concept. Adapted from Persson (2021).

However, a recent study by Dewitt et al. (2021) found that physicians, nurses and OTs primarily understand proven experience in terms of interventions/methods/etc. *being tested*, in terms of *being part of accepted practice* and in terms of professionals or teams of professionals *being experienced (see also* Fig. 1*)*.

### 2.2 Science and proven experience in higher education

**Fig. 2:**
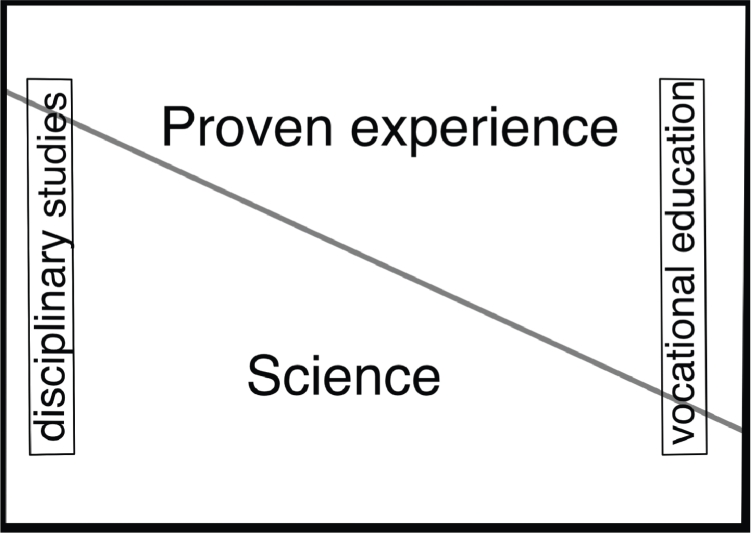
Differences in the proportion of scientific knowledge and proven experience in the curricula of various higher educations; traditional academic disciplinary studies to the left and higher vocational education to the right. (Figure adapted from Prop. Regeringens proposition 1992/93: 1992, Utbildningsutskottets betänkande 1992, p. 26).

There is one other important source to consider when pondering VBE. The preparatory works leading to the legal requirement that all higher education in Sweden – except in the performing arts – should be based (albeit to varying degree) on both science and proven experience, provides an illustration of how science and proven experience is supposed to interrelate in higher education, thus providing the basis for their interrelation in the professionals’ future decisions. In preparation for the new legislation the government proposed the schema in Fig. 2.

Assuming the model in Fig. 2, the knowledge base of traditional academic disciplines has less practice-derived knowledge than that of vocational education. From the consultation responses to the government's preparatory works it became clear that several healthcare educations, in particular, were perceived as belonging to the right hand side of the spectrum in Fig. 2, i.e. containing a large amount of proven experience (see e.g. 1992/93:UBU03), with medicine further to the left than nursing.

**Fig. 3:**
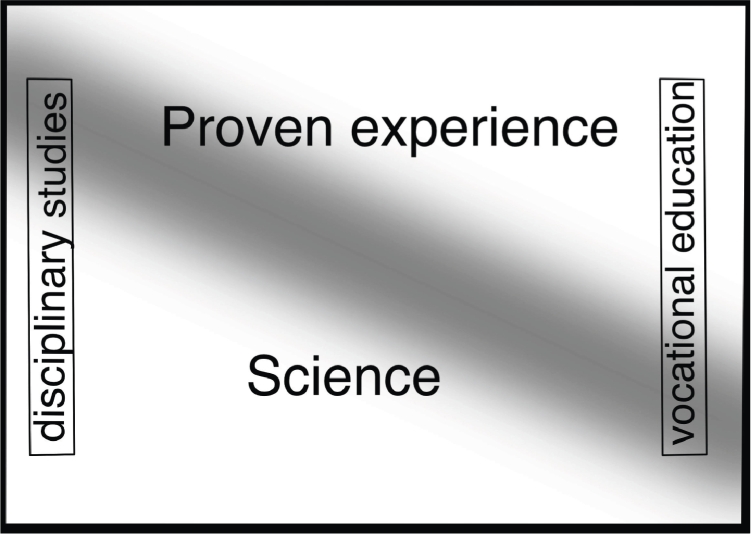
Differences in the proportion of scientific knowledge and proven experience in the curricula of various higher educations; traditional academic disciplinary studies to the left and higher vocational education to the right. The Gaussian blur represents parts of the curriculum that builds on knowledge harboured *both* in science *and* proven experience.

As far as we understand, the representation in Fig. 2 is not normative but tries to be descriptive. In that way it provides *prima facie* information about the dynamics we set out to probe in this paper. From the representation we can read off a preliminary answer to some of our questions. Most importantly, it seems to be suggested that practice-derived knowledge – especially in vocational education – can sometimes provide a knowledge base in a similar way that scientific evidence can. However, an obvious objection to that claim is that the representation in Fig. 2 is overly simple. It does not incorporate a situation where an item of the curriculum is part of *both* science *and* proven experience. If we assume that the problem is not with the visualization but with the curricula, further curricular development of practice-derived knowledge in the more traditional academic disciplines and theoretical advances in higher vocational education is required to meet the legal requirement. A more satisfactory balance would be reached in a situation such as represented in Fig. 3.

Presumably, if the representation in Fig. 3 is preferable to the one in Fig. 2 for descriptive purposes,then it seems to follow that practice-derived knowledge in the curriculum can have two roles. One *complementary* role (when there is not enough science to base the education on) and one for *integrative* purposes (when the content is highlighted from two evidential sources). The complementary role can be acknowledged in both VBE and EBM, but in EBM the complementary role is present only in the *absence* of scientific evidence, whereas in VBE the absence of scientific evidence is a special case.

The difference between EBM and VBE is visible also within the integrative role. The integrative aspect is fundamental in EBM for *implementation* purposes (Dewitt et al. 2021); the clinician needs to understand *how* to apply the evidence-based knowledge in a concrete case. But in those cases practice-derived knowledge is not evidence, at least not clearly – it is rather knowledge how or knowledge how to implement. In VBE, on the other hand, the integrative use (the Gaussian blur in Fig. 3) is central. In VBE, the integrative use does not have to be motivated by the need for implementation or knowledge *how*; it can still be knowledge *that* one wants to strengthen – and for this reason practice-derived knowledge is perceived as evidence also in the integrative use. In a sense, whether VBE results in practice-derived knowledge being complementary or integrative is a consequence of whether science and proven experience converge or diverge and not a consequence of a variation in the nature of practice-derived knowledge as such.

## 3 Hypotheses

When two sources of evidence, such as scientific and practice-derived evidence are compared or integrated, they need to be comparable on similar dimensions. Similarly, when one of them is to compensate for the lack of the other. In other words, for compensatory and integrative uses (but not necessarily for implementation purposes) similarities in nature between the two should be required.

For decision making the *certainty* of evidence matters. A large part of the evidence hierarchies provided by, for instance, GRADE, aims at providing users with a sense of how certain available scientific evidence is. Comparing evidence types based on their certainty is therefore to be expected when both scientific evidence and practice-derived evidence are taken into account for integrative or compensatory reasons. From this, it should follow that *certain* proven experience is perceived to be more important for decision-making purposes than less certain proven experience (or less certain scientific evidence) *if practice-derived evidence has a compensatory or integrative role, which is required by VBE but not – in general – by EBM*.

Moreover, on the plausible assumption that – within healthcare professions – scientific evidence is the ideal, due to the influence of EBM and its forerunners, we should expect to find that, as long as proven experience is judged certain enough it should also be deemed important for decision-making.

There are thus reasons why the following hypothesis is to be expected to hold if the compensatory and integrative roles laid out in VBE are descriptively right:

*H1: The more certain proven experience is perceived to be, the more important is it perceived to be for professional decision-making*

However, practical decision-making is seldom characterised in terms of certainty (but see Levi (1971)). It typically takes place under uncertainty or even ignorance. Moreover, a distinction between knowing why and knowing how is often applicable when comparing the two sources of evidence (see e.g Brante 2010). There is a difference between knowing why a drug is effective and how to administer it safely and effectively. Whether a particular treatment is acceptable to the patient, and how it should be implemented requires sound judgment, based on clinical expertise and knowledge how. H1 is therefore not a trivial hypothesis. It involves the conjecture that even if proven experience has evolved to manage situations where we have to rely on, for instance, coarse-grained clinical knowledge, typically depending on unknown characteristics of the local context, the certainty of such knowledge matters to the professionals.

For partly similar reasons we should, in the same circumstances, expect that more *systematic* proven experience results in more important proven experience:

First, and plainly, with certain systematicity in knowledge it is easier to frame a general strategy for how that kind of knowledge is to be taken into account. Indirectly, more systematic evidence reduces uncertainty, as it will be more clear when it is applicable and when it is not.

Second, science is characterised by being systematic. For instance, Hoyningen-Huene (2013) argues that science is more systematic than everyday knowledge in nine ways: in its descriptions, explanations, predictions, defense of knowledge claims, critical discourse, epistemic connectedness, ideal of completeness, knowledge generation, and representation of knowledge. To the extent that scientific evidence is the ideal, proven experience that develops towards more systematicity should be perceived more important:

*H2: The more systematic proven experience is perceived to be, the more important it is perceived to be.*

Again, H2 is interesting from the point of view of EBM in that the hypothesis is not strongly supported through the implementation role assigned to practice-derived knowledge.

In fact, unless integrative and compensatory mechanisms were in place, we would not expect that scientific evidence and practice-derived knowledge should put the same emphasis on certainty and systematicity. Medical decisions are normally made in the face of multiple, often conflicting factors and for individual cases implying that certainty is often not a realistic goal for practice-derived knowledge. Moreover, practice-derived knowledge might be expected never to be more than basically systematic, given the unpredictability of who appears for the professional to help.

An additional set of hypotheses along these lines are more exploratory. In order to probe if the balance between science and proven experience is in fact affected by the development of proven experience in such a way that the difference in importance of the two diminishes, the following hypotheses were formulated:

*H3: The more certain proven experience is perceived to be, the smaller the perceived difference between the importance of science and proven experience becomes (regardless of direction).*

*H4: The more systematic proven experience is perceived to be, the smaller the perceived difference between the importance of science and proven experience becomes (regardless of direction).*

Finally, even if this is perhaps not to be expected in professions with an already well-functioning scientific evidence base, and presumably never within the EBM framework, it is not impossible that sometimes increased certainty or systematicity of proven experience is negatively correlated with the perceived importance of science.

At least within a VBE-context, when scientific and practice-derived evidence are balanced against each other, we would expect professions with less definite relations to specific sciences to rely more on proven experience. We do not know which professions have stronger, more robust ties to science. However, it seems plausible that the strength of a profession's tie to a certain, systematic science is a function of time. Since most healthcare professions have considerable historical roots, placing the professions in an approximate order of professional age is not trivial. But medicine, dentistry, nursing, OT, and dental hygiene is arguably approximately the right order. Nursing is interesting. It is not the oldest profession – in an academic/legal sense – but the scientific and professional status of nursing has been in focus since the 19th century.

Since both certainty and systematicity are salient features of science we might expect that in the short run it is exactly when such features are promoted in the development of proven experience that the importance of scientific evidence might decrease:

*H5: The more certain proven experience is perceived to be, the less important science for clinical decision making becomes.*

*H6: The more systematic proven experience is perceived to be, the less important science for clinical decision-making becomes.*

## 4 Methods: the design of the study and the analysis

In order to study the relationships between scientific evidence and proven experience we conducted two surveys directed at five healthcare professions of varying professional ages: medicine/physicians, dentists, nurses, occupational therapists, and dental hygienists.

### 4.1 Study design

A survey was set to simple random samples of Swedish physicians, nurses, occupational therapists, dentists, and dental hygienists. It was administered by the government statistical agency: Statistics Sweden, in two waves. In 2018 physicians, nurses and occupational therapists were contacted, and in 2019 we reached out to dentists and dental hygienists. As a sampling frame, the most recent occupational registries were used. For the 2018 survey (physicians, nurses and occupational therapists) the registry was from 2015, and the sampling frame consisted of 33 618 physicians, 91 174 nurses and 7 412 occupational therapists. For the 2019 survey the registry was from 2017 and the sampling frame consisted of 6 155 dentists and 3 699 dental hygienists. For both waves, 700 members of each profession were contacted. The study design was approved by Lund University Regional Ethics Board (decision dnr 2017/428). Participants were not compensated.

All participants were first contacted by mail consisting of study information and log-in to a web-based survey (January 2018 for the first wave and October 2019 for the second wave; number of responses 379 and 249 respectively). A second contact was made by mailing a paper-based version of the survey to participants (resulted in 299 resp. 201 additional responses). The third and last contacts involved a reminder (132 and 100 responses resp.) and finally a second paper-based survey (170 resp 97 responses). Data collection closed April 2018 for the first wave, and January 2020 for the second wave.

The survey consisted mostly of questions about participants' beliefs about and attitudes towards proven experience. A full translation can be found in the appendix. This paper is based on participants’ responses to three questions concerning the importance, certainty and systematicity of proven experience and other types of evidence. In particular, participants were asked:

1) How important they think proven experience and scientific evidence are for sound decision making in healthcare. Answers were given on a five-point Likert scale with the end points “not at all important” and “very important.”
2) How certain they think knowledge from proven experience and scientific evidence are in healthcare. Answers were given on a five-point Likert scale with the end points “not at all certain” and “very certain.”
3) How systematic they think knowledge from proven experience and scientific evidence respectively are in healthcare. Answers were given on a five-point Likert scale with the end points “not at all systematic” and “very systematic.”

## 5 Results

A total of 1626 surveys were returned. A total of 26 participants were removed prior to analysis (the exclusion criteria were: more than one profession indicated, missing certificate, and mistake in stratum). The final sample consisted of 1600 responses out of 3500 surveys distributed: 295 physicians, 300 nurses, 365 occupational therapists, 339 dentists, and 301 hygienists. Furthermore, 162 responses in questions used as variables in the analyses were either uninterpretable or empty; those were replaced with the modal response for a given participant's profession on a given question.

**Fig. 4:**
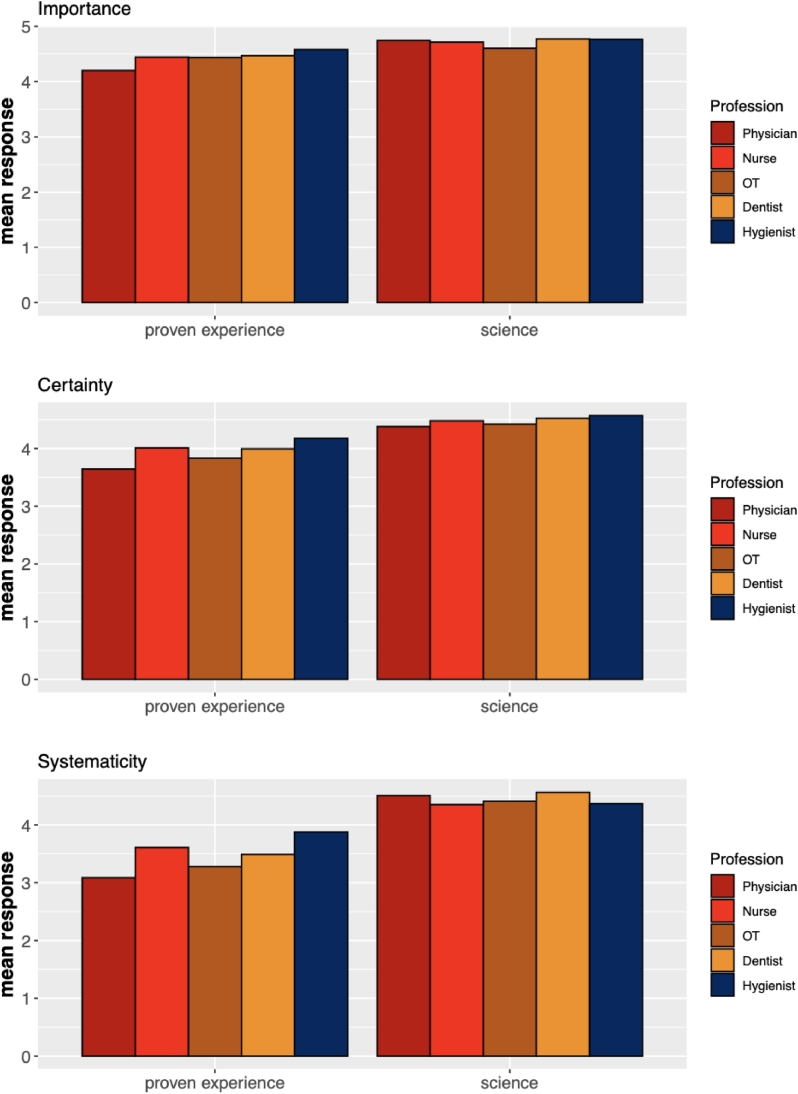
Perceived importance, certainty and systematicity of proven experience and science among physicians, dentists, nurses, OTs, and dental hygienists.

In our study the importance, certainty, and systematicity of science was uncontested. In all five healthcare professions we studied the mean values of the importance, certainty, and systematicity of scientific evidence were generally higher for scientific evidence than for proven experience (see Fig. 4). This is certainly in line with EBM and also with VBE. However, the mean responses concerning the importance of proven experience also score high, particularly among professionals other than physicians. This is consistent with both EBM and VBE, but entailed by VBE.

We report the results for the hypotheses, H1-H6, by grouping the hypotheses into three categories based on common dependent variables for their corresponding models: H1 and H2 measure the importance of proven experience; H3 and H4 measure the difference in importance between science and proven experience; and H5 and H6, finally, measure the importance of science.

H1 and H2:

The results of the tests of H1 and H2 are shown in Table 1.

**Table 1:** Regression results for hypotheses 1 and 2.

	Dependent variable:	
	Importance of proven experience	
	(1)	(2)
Systematicity (PE)	^0.364^∗∗∗ ^(0.283,0.444)^	
Certainty (PE)		0.537∗∗∗ (0.458,0.616)
Nurse	0.247∗ (−0.028,0.522)	0.215 (−0.111,0.541)
OT	0.659∗∗∗ (0.401,0.917)	0.579∗∗∗ (0.259,0.898)
Dentist	0.365∗∗∗ (0.110,0.619)	0.298∗ (−0.027,0.623)
Hygienist	0.313∗ (−0.003,0.629)	0.413**(0.050,0.775)
Systematicity (PE):Nurse	−0.074 (−0.183,0.035)	
Systematicity (PE):OT	−0.215∗∗∗ (−0.325,−0.105)	
Systematicity (PE):Dentist	−0.097∗ (−0.201,0.007)	
Systematicity (PE):Hygienist	−0.076 (−0.194,0.042)	
Certainty (PE):Nurse		−0.056 (−0.167,0.055)
Certainty (PE):OT		−0.156∗∗∗ (−0.268,−0.044)
Certainty (PE):Dentist		−0.072 (−0.183,0.039)
Certainty (PE):Hygienist		−0.100∗ (−0.219,0.019)
Constant	3.441*** (3.259,3.623)	2.780*** (2.561,2.999)
Observations	1600	1600
_R_^2^	0.166	0.311
Adjusted R^2^	0.161	0.307
Residual Std. Error (df = 1590)	0.618	0.562
F Statistic (df = 9; 1590)	35.194∗∗∗	79.683∗∗∗

Note:.

∗∗*p* < 0.05.

∗ *p* < 0.1.

∗∗∗ *p* < 0.01.

As shown in Table 1, H1 and H2 bare out for physicians, and also for the other professions, but to a lesser degree. The interaction, showing how the effect on physicians is heightened/lessened for the other professions indicate that the effect is always lessened, but not to the extent that the hypothesis is contradicted. The importance of proven experience is clearly dependent on its certainty and systematicity.

H3&H4:

The results of the tests of H3 and H4 are shown in Table 2.

**Table 2:** Regression results for hypotheses 3 and 4.

	Dependent variable:	
	Difference betw. importance of proven experience and science	
	(1)	(2)
Certainty (PE)	0.537∗∗∗ (0.439,0.636)	
Systematicity (PE)		0.391∗∗∗ (0.296,0.487)
Nurse	0.391∗ (−0.015,0.797)	0.367∗∗ (0.040,0.695)
OT	0.790∗∗∗ (0.391,1.189)	0.834∗∗∗ (0.527,1.141)
Dentist	0.339 (−0.067,0.745)	0.442∗∗∗ (0.139,0.745)
Hygienist	0.960∗∗∗ (0.508,1.413)	0.617∗∗∗ (0.241,0.992)
Certainty (PE):Nurse	−0.105 (−0.243,0.033)	
Certainty (PE):OT	−0.181∗∗ (−0.321,−0.041)	
Certainty (PE):Dentist	−0.095 (−0.234,0.043)	
Certainty (PE):Hygienist	−0.279∗∗∗ (−0.428,−0.131)	
Systematicity (PE):Nurse		−0.115∗ (−0.245,0.015)
Systematicity (PE):OT		−0.233∗∗∗ (−0.364,−0.102)
Systematicity (PE):Dentist		−0.144∗∗ (−0.268,−0.019)
Systematicity (PE):Hygienist		−0.197∗∗∗ (−0.337,−0.056)
Constant	−1.963∗∗∗ (−2.236,−1.690)	−1.359∗∗∗ (−1.576,−1.143)
Observations	1600	1600
_R_^2^	0.200	0.120
Adjusted R^2^	0.196	0.115
Residual Std. Error (df = 1590)	0.701	0.735
F Statistic (df = 9; 1590)	44.226∗∗∗	24.161∗∗∗

Note:.

∗ *p* < 0.1.

∗∗ *p* < 0.05.

∗∗∗ *p* < 0.01.

Again, as can be seen in Table 2, the results for physicians are as hypothesized, with the other professions showing the same relationship, but lessened in magnitude. Of course, because all the responses are on bounded scales, the theoretical maximum rate of change is constrained. Professions other than physicians start out seeing less of a difference between the two types of knowledge, so there is less room to show the hypothesized effect in those professions.

H5&H6:

The tests of H5 and H6 are shown in Table 3.

**Table 3:** Regression results for hypotheses 5 and 6.

	Dependent variable:	
	Importance of science	
	(1)	(2)
Systematicity (PE)	−0.028 (−0.099,0.044)	
Certainty (PE)		−0.0003 (−0.078,0.077)
Nurse	−0.121 (−0.367,0.125)	−0.176 (−0.495,0.143)
OT	−0.175 (−0.406,0.055)	−0.211 (−0.524,0.102)
Dentist	−0.077 (−0.305,0.151)	−0.041 (−0.360,0.278)
Hygienist	−0.304∗∗ (−0.586,−0.021)	−0.547∗∗∗ (−0.903,−0.192)
Systematicity (PE):Nurse	0.041 (−0.057,0.139)	
Systematicity (PE):OT	0.018 (−0.080,0.117)	
Systematicity (PE):Dentist	0.046 (−0.047,0.140)	
Systematicity (PE):Hygienist	0.121∗∗ (0.015,0.226)	
Certainty (PE):Nurse		0.049 (−0.060,0.157)
Certainty (PE):OT		0.025 (−0.085,0.135)
Certainty (PE):Dentist		0.023 (−0.086,0.132)
Certainty (PE):Hygienist		0.179∗∗∗ (0.063,0.296)
Constant	4.800∗∗∗ (4.638,4.963)	^4.743^∗∗∗ (4.529,4.957)
Observations	1600	1600
_R_^2^	0.017	0.025
Adjusted R^2^	0.012	0.019
Residual Std. Error (df = 1590)	0.553	0.550
F Statistic (df = 9; 1590)	3.141∗∗∗	4.471∗∗∗

Note:.

∗*p* < 0.1.

∗∗ *p* < 0.05.

∗∗∗ *p* < 0.01.

As Table 3 shows, the coefficients are in the hypothesized direction, but they are small, and not statistically significant – except for hygienists. One reason could be that there is not much variance in the outcome variable – 5 is the modal (and median) response for every profession. If nearly everyone thinks science is extremely important, then nothing else (e.g., ratings of the certainty of proven experience) will covary much with it, because there is so little variance in the dependent variable. Science **is** considered extremely important by all, irrespective of the certainty and systematicity of relevant proven experience, but for dental hygienists proven experience is also very important.

## 6 Discussion

Before this study we knew very little about the perceived relative weight of practice-derived evidence and the balance or dynamics between scientific evidence and practice-derived knowledge among healthcare professions. We have studied that question within the context of the Swedish concept ‘science and proven experience,’ which regulates healthcare practice as well as education in the country. It is to be expected that the normative/legal notion of VBE has affected the perceptions of Swedish professionals given they are subject to regulation defined by laws. There are also examples of Swedish physicians stating that EBM is just a reformulation of the old Swedish motto (see e.g Werkö 2000., Werkö et al. 2002), which potentially makes the comparison even more interesting for EBM development purposes (Persson et al. 2019).

The evidence on which a professional builds her decisions can belong to several epistemic domains: it can be a piece of personal experience, proven experience or scientific evidence. Most of the time, evidence from all three domains combine to inform the healthcare professional's decisions. The exact proportion of each likely varies throughout the professional's career, from mostly science at the beginning (when personal experience is limited), to mostly experience at the end (when, unless continuing education is effective, experience is paramount).

Scientific evidence is clearly highly valued within the healthcare professions. It is perceived as important, certain, and systematic (see Fig. 4). But proven experience is also highly valued.

It is clear from the results that proven experience's perceived importance for clinical decision-making is positively correlated with its certainty and systematicity (Table 1). It is also clear that its increased certainty and systematicity is positively correlated with a diminished difference in importance between science and proven experience for almost all professions surveyed in this study (Table 2). Those results indicate that proven experience has an evidentiary role in clinical decision-making, and that this role depends in part on its certainty and systematicity. That is interesting from an EBM perspective as it makes the take on practice-derived knowledge as being primarily of implementation value less plausible.

These correlations hold for the entire wide array of health care professions we have studied. For dental hygienists, moreover, the importance of science for professional decision-making *decreases* with increases in both certainty and systematicity of proven experience (Table 3). This is interesting in itself. It might for instance be that this is explained by the fact dental hygiene is among the youngest professions in our study. It is plausible that young professions are less inextricably reliant on science. These results might thus corroborate the presence of a dynamic in young professions balancing science and proven experience in such a way that the absence of unique, certain, and systematic scientific grounding is compensated for by more certain and systematic proven experience. Here we might get an indication that lack of a strong relation to a scientific theory has the consequence that compensatory attempts to strengthen the proven experience of the profession, making it more certain and systematic, might take place. Such a dynamic would be difficult to reconcile with EBM.

*Prima facie* It is an open question whether proven experience and scientific knowledge will converge or diverge (Persson 2021). The positive correlation between proven experience's importance and systematicity arguably points in the direction of convergence. It is unrealistic that two systematic but non-overlapping bodies of evidence providing answers to the same question should evolve. It thus seems plausible that the convergence thesis is right where professions have strong links to scientific evidence.

It might still be a real possibility that we will see divergence between proven experience and scientific knowledge in professions with weak relations to scientific evidence. Whether this would be desirable or unfortunate is not for this paper to say. It would probably be unfortunate, in the short run at least, for the sciences. They would lose some of their relevance. However, it is possible that the emergence of certain, systematic proven experience would be a rich source of knowledge for future science to feed from – and thus that a temporary divide might be closed at a later stage.

## 7 Conclusions

The data in this study strongly confirmed that balancing with regard to certainty, systematicity, and importance takes place between practice-derived knowledge – operationalized here as the Swedish concept of “proven experience”– and scientific evidence among health care professionals belonging to medicine, nursing, dentistry, OT, and dental hygiene. That such a dynamic should exist in Sweden is not surprising given that the normative/legal notion of science and proven experience (VBE) was articulated and has governed actual practice in Swedish medicine for more than a century. It is partly more surprising from an EBM perspective (which has also been extremely influential in Sweden and everywhere else), particularly because the pattern is strong in the profession with the most well-developed scientific basis (medicine). EBM typically interprets the role of practice-derived knowledge in such cases as implementation knowledge, and this does not seem entirely coherent with the results. Furthermore, both certainty and systematicity of practice-derived evidence was shown to affect the importance of proven experience as evidence for health care decisions among healthcare professionals.

## 8 Funding sources

Work on this paper was supported by a grant from the program on Science and Proven Experience (Vetenskap och Beprövad Erfarenhet) administered by the Swedish Foundation for Humanities and Social Sciences (Riksbankens Jubileumsfond), grant no. M14–0138:1.

## 9 Data availability statement

: The dataset containsdata that qualifies as personal data under the EU General Data Protection Regulation (GDPR). Legal restrictions for accessing and transferring the data apply. Therefore, the data cannot be made fully available. The data is deposited at Lund University, and can be accessed by anyone with a legitimate interest in it by sending an inquiry to registrator@lu.se, to the extent that the transfer of data is in accordance with the applicable legal regulation. During the research process the authors have had access to the data. The authors do not have any special access privileges that others would not have. (Fig. 1)

## Declaration of Competing Interest

None

## Appendix Supplementary materials

Image, application 1

## Footnotes

1 It should be noted that not everyone agrees with this characterisation Karthikeyan and Pais (2010). state that: “EBM only requires that the clinician be sufficiently familiar with the evidence-base in his/her field and be able to objectively appraise it, so that he or she can apply it appropriately in practice.”

2 For an empirical analysis of the meaning and use of “proven experience”, see Dewitt et al. (2021) and Persson and Wahlberg (2015).
