# Supplementary material for: Certainty and systematicity of practice-derived evidence matter for its relative importance in professional decision-making: Survey results on the role of proven experience in Swedish medicine, nursing, OT, dentistry, and dental hygiene
Source: Int J Nurs Stud Adv. 2022 Mar 17;4:100074. doi: 10.1016/j.ijnsa.2022.100074 (PMC11080282; doi:10.1016/j.ijnsa.2022.100074)
Supplement: Supplementary file 1 [file mmc1.docx]

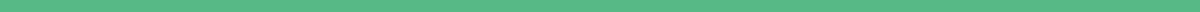


Supporting information

# OVERVIEW

This document provides supporting information for the paper, “Certainty and systematicity of practice-derived evidence matter for its relative importance in professional decision-making The role of proven experience in Swedish medicine, nursing, OT, dentistry, and dental hygiene”

First, we present the original version of the survey given to the first wave of participants in the study (it was edited for consistency and relevance in the second wave, but these editions do not affect the subset of question prompts (F19-F21) used in this paper. Second, we include a statement of how results from other parts of the study have been used by the authors.

# Survey

Below is an English translation of the survey administered to the first wave of the study’s participants. The English translation was created by the authors, but all participants took the survey in the original Swedish. The broader aim of the survey was to understand clinicians’ views of their own expertise, and in particular how they understood the legal notions “science and proven experience” governing their work. In this particular paper we target clinicians’ view of the legal role of the notion.

**Proven experience in communication between colleagues**

**A.** First two questions about how well the concept of "proven experience" works in the communication between colleagues.

**1. How certain do you feel that you know what the term "proven experience" means when you use the term?**

| 1 | 2 | 3 | 4 | 5 |
| --- | --- | --- | --- | --- |
| Not at all certain |  |  |  | Completely certain |

If you want to, please feel free to comment on your answer here: [Free text option]

**2. How certain do you think that your colleagues in healthcare generally feel about what the term "proven experience" means when they use the term?**

| 1 | 2 | 3 | 4 | 5 |
| --- | --- | --- | --- | --- |
| Not at all certain |  |  |  | Completely certain |

If you want to, please feel free to comment on your answer here: [Free text option]

**How do you perceive proven experience?**

**B.** In this section of the questionnaire there is a number of statements about proven experience. Please respond based on how you perceive proven experience, regardless of whether it is consistent with how you think others perceive the term.

**3. That there is proven experience of a treatment in the field of health care means that it has been carefully tested in the field of health care.**

| 1 | 2 | 3 | 4 | 5 |
| --- | --- | --- | --- | --- |
| Do not agree at all |  |  |  | Completely agree |

**4. That there is proven experience of a treatment in health care means that it has been shown to be effective in the field of healthcare.**

| 1 | 2 | 3 | 4 | 5 |
| --- | --- | --- | --- | --- |
| Do not agree at all |  |  |  | Completely agree |

**5. That there is proven experience of a treatment in health care means that a group of healthcare professionals together have reached the conclusion that it works.**

| 1 | 2 | 3 | 4 | 5 |
| --- | --- | --- | --- | --- |
| Do not agree at all |  |  |  | Completely agree |

**6. That there is proven experience of a treatment in health care means that it is widely accepted among healthcare professionals.**

| 1 | 2 | 3 | 4 | 5 |
| --- | --- | --- | --- | --- |
| Do not agree at all |  |  |  | Completely agree |

**7. That there is proven experience of a treatment in health care means that its origins lie in the daily activities in health care.**

| 1 | 2 | 3 | 4 | 5 |
| --- | --- | --- | --- | --- |
| Do not agree at all |  |  |  | Completely agree |

**8. That there is proven experience of a treatment in health care means that it does not violate medical ethics.**

| 1 | 2 | 3 | 4 | 5 |
| --- | --- | --- | --- | --- |
| Do not agree at all |  |  |  | Completely agree |

**9. That there is proven experience of a treatment in health care means that it has been used by many health care professionals.**

| 1 | 2 | 3 | 4 | 5 |
| --- | --- | --- | --- | --- |
| Do not agree at all |  |  |  | Completely agree |

**10. Healthcare professionals can have proven experience of carrying out a medical measure.**

| 1 | 2 | 3 | 4 | 5 |
| --- | --- | --- | --- | --- |
| Do not agree at all |  |  |  | Completely agree |

**11. That there is proven experience of a treatment in health care means that it works in the day-to-day activities in health care.**

| 1 | 2 | 3 | 4 | 5 |
| --- | --- | --- | --- | --- |
| Do not agree at all |  |  |  | Completely agree |

**12. That there is proven experience of a treatment in health care means that it is used by healthcare professionals for the current purpose.**

| 1 | 2 | 3 | 4 | 5 |
| --- | --- | --- | --- | --- |
| Do not agree at all |  |  |  | Completely agree |

**13. Proven experience is obvious to anyone who has a lot of experience in the profession.**

| 1 | 2 | 3 | 4 | 5 |
| --- | --- | --- | --- | --- |
| Do not agree at all |  |  |  | Completely agree |

**14. That there is proven experience in treatment means that it is based on the professional’s common sense.**

| 1 | 2 | 3 | 4 | 5 |
| --- | --- | --- | --- | --- |
| Do not agree at all |  |  |  | Completely agree |

**15. Proven experience in health care includes experience of what patients prefer.**

| 1 | 2 | 3 | 4 | 5 |
| --- | --- | --- | --- | --- |
| Do not agree at all |  |  |  | Completely agree |

**16. That there is proven experience of a treatment in health care means that it is used by successful medical units.**

12345

Do not agree at all – Completely agree

**17. That there is proven experience of a treatment in health care means that there is information documented about what has happened when it has been used.**

| 1 | 2 | 3 | 4 | 5 |
| --- | --- | --- | --- | --- |
| Do not agree at all |  |  |  | Completely agree |

**18. That there is proven experience of a treatment in health care means that it has been used in health care for a long time.**

| 1 | 2 | 3 | 4 | 5 |
| --- | --- | --- | --- | --- |
| Do not agree at all |  |  |  | Completely agree |

**Proven experience and other types of evidence**

**C.** In this part of the survey, there is a number of claims about how proven experience relates to other knowledge and evidence in health care.

**19.How important are each of the following types of knowledge for sound decision making in the healthcare sector?**

**Personal experience**

| 1 | 2 | 3 | 4 | 5 |
| --- | --- | --- | --- | --- |
| Not at all important |  |  |  | Very  important |

**Proven experience**

| 1 | 2 | 3 | 4 | 5 |
| --- | --- | --- | --- | --- |
| Not at all important |  |  |  | Very  important |

**Scientific evidence**

| 1 | 2 | 3 | 4 | 5 |
| --- | --- | --- | --- | --- |
| Not at all important |  |  |  | Very  important |

**20. How certain are each of the following types of knowledge in healthcare?**

**Personal experience**

| 1 | 2 | 3 | 4 | 5 |
| --- | --- | --- | --- | --- |
| Not at all certain |  |  |  | Completely certain |

**Proven experience**

| 1 | 2 | 3 | 4 | 5 |
| --- | --- | --- | --- | --- |
| Not at all certain |  |  |  | Completely certain |

**Scientific evidence**

| 1 | 2 | 3 | 4 | 5 |
| --- | --- | --- | --- | --- |
| Not at all certain |  |  |  | Completely certain |

**21. How systematic are each of the following types of knowledge in the healthcare system?**

**Personal experience**

| 1 | 2 | 3 | 4 | 5 |
| --- | --- | --- | --- | --- |
| Not at all systematic |  |  |  | Very systematic |

**Proven experience**

| 1 | 2 | 3 | 4 | 5 |
| --- | --- | --- | --- | --- |
| Not at all systematic |  |  |  | Very systematic |

**Scientific evidence**

| 1 | 2 | 3 | 4 | 5 |
| --- | --- | --- | --- | --- |
| Not at all systematic |  |  |  | Very systematic |

**The legal requirement of science and proven experience**

**D.** In this section of the survey, a few questions follow on how you perceive the requirement of science and proven experience in the legal regulation of healthcare.

According to the Patient Act, patients shall receive healthcare that is in accordance with science and proven experience. The Patient Safety Act states that healthcare professionals have a personal responsibility to carry out their work in accordance with science and proven experience.

**22. How certain do you feel that you know what the term "science and proven experience" means in the legal regulation of healthcare?**

| 1 | 2 | 3 | 4 | 5 |
| --- | --- | --- | --- | --- |
| Not at all certain |  |  |  | Completely certain |

**23. How do you feel about "science and proven experience" being used as quality requirement in the legal regulation of healthcare?**

| 1 | 2 | 3 | 4 | 5 |
| --- | --- | --- | --- | --- |
| Not at all satisfied |  |  |  | Very satisfied |

If you want to, please feel free to comment on your answer here: [Free text option]

**24. How do you feel about "proven experience" being used as quality requirement in the legal regulation of healthcare?**

| 1 | 2 | 3 | 4 | 5 |
| --- | --- | --- | --- | --- |
| Not at all satisfied |  |  |  | Very satisfied |

If you want to, please feel free to comment on your answer here: [Free text option]

**25. Who should - in your opinion - determine how the term "science and proven experience" should be interpreted in the legal regulation of healthcare?**

*You can choose more than one option if you want.*

Doctors and nurses and others in the healthcare professions

Courts / lawyers

SBU/Socialstyrelsen/IVO

Those who health care related research

Don’t know

If something else, please describe it here: [Free text option]

**Some questions about you**

**E.** At last,some questions about who you are.

**26. How old are you**

25 years or younger

26-30

31-35

36-40

41-45

46-50

51-55

56-60

61-65

66 years or older

**27. I am**

Woman

Man

I cannot / do not want to use the options above

**28. What are you currently employed as?**

Nurse

Doctor

Occupational therapist

If something else, please specify: [Free text option]

**29. If you have any specialization, which one is it?**

[Free text]

**30. How long have you had this employment?**

0-5 years

6-10 years

11-15 years

16-20 years

21-25 years

26-30 years

More than 30 years

**31. How long is it since you got your certification**

0-5 years

6-10 years

11-15 years

16-20 years

21-25 years

26-30 years

More than 30 years

**32. Where did you get your license?**

Göteborg

Köpenhamn

Lund

Stockholm

Umeå

Uppsala

Växjö

If something else, please specify: [Free text option]

**33. What academic degree do you have?**

[Free text option]

#

# RELATED WORK

Defining the study in terms of the two surveys we have conducted, the following publications and planned publications result from the study. However, it should be noted that there is only a very slight overlap in the data used. The first paper is:

Wallin, A, Wahlberg, L, Persson, J & Dewitt, B 2020, '“Science and proven experience”: How should the epistemology of medicine inform the regulation of healthcare?', Health Policy, vol. 124, no. 8. https://doi.org/10.1016/j.healthpol.2020.05.005

It concentrates on the responses to question prompts on how certain professionals are about the meaning of “proven experience” (Q1, Q2) and how satisfied they are with the legal notion (Q22-Q25). Also, it only contains data from one of the two surveys.

The second paper is:

Dewitt B, Persson J, Wahlberg L, Wallin A. 2021. The epistemic roles of clinical expertise: An empirical study of how Swedish healthcare professionals understand proven experience. PLoS ONE 16(6): e0252160. https://doi.org/10.1371/journal.pone.0252160

It uses responses on the question prompts in the survey corresponding to the conceptual aspects of proven experience (Q3-Q18). These responses are not used at all in the submitted paper. Also, it only contains data from one of the two surveys. However, the concept of proven experience is touched on, with a reference to this paper.

The third paper builds on the same data in the sense that it reports the mean values for all responses, as in Figure 4 in the present paper (overlapping data), but it does not use the data in any further analysis. It is a Swedish professional journal and not read by an international audience. Hence it is not referred to in the submitted paper:

Sahlin, N-E, Dewitt, B, Persson, J, Wahlberg, L & Wallin, A. 2020. 'Så uppfattar tandläkare beprövad erfarenhet: några resultat från en enkätstudie', Tandläkartidningen, vol. 2020, no. 7, pp. 50-54. <https://www.tandlakartidningen.se/wp-content/uploads/2020/06/Sahlin-et-al_Webbversion.pdf>
